# G Protein-Coupled Receptor 30 Attenuates Neuronal Pyroptosis Induced by Subarachnoid Hemorrhage

**DOI:** 10.1155/mi/3585885

**Published:** 2025-07-21

**Authors:** Jun Peng, Xiqi Hu, Jun He, Ying Xia

**Affiliations:** Department of Neurosurgery, Haikou Affiliated Hospital of Central South University Xiangya School of Medicine, Haikou, China

**Keywords:** IFI16, inflammasome, NLRC4, pyroptosis, subarachnoid hemorrhage, TLR4/NF-κB

## Abstract

**Background:** Pyroptosis is implicated as a pathogenic mechanism in early brain injury (EBI) after subarachnoid hemorrhage (SAH). This study aimed to investigate the regulatory role of G protein-coupled receptor 30 (GPR30) in neuronal pyroptosis during SAH.

**Methods:** SAH was induced in rats via intravascular perforation and hemin-treated neurons modeled SAH in vitro. GPR30 agonist G1 and antagonist G15 were administered to assess functional impacts. Neurological deficits (Garcia score), SAH severity, and cerebral edema (brain water content) were evaluated. Pyroptotic markers (cleaved caspase-1, gasdermin D (GSDMD)-N, interleukin (IL)-1β, and IL-18) were quantified. Inflammasome activation (NLRC4 and IFI16) and Toll-like receptor 4/nuclear factor kappa-B (TLR4/NF-κB) signaling were analyzed via immunofluorescence (IF) and immunoblotting. The TLR4 antagonist LPS-RS (lipopolysaccharide from *Rhodobacter sphaeroides*) was applied to validate pathway involvement.

**Results:** GPR30 expression increased post-SAH. G15 exacerbated hemorrhage severity, neurological deficits, and cerebral edema, whereas G1 modestly attenuated SAH. G15 upregulated pyroptotic markers, enhanced neuronal pyroptosis, and activated NLRC4/IFI16 inflammasomes. Concurrently, G15 stimulated TLR4/MyD88 expression and NF-κB phosphorylation. Conversely, G1 suppressed pyroptosis, inactivated inflammasomes, and inhibited TLR4/NF-κB signaling. LPS-RS cotreatment reversed G15-induced pyroptotic and inflammatory cascades.

**Conclusion:** GPR30 mitigates NLRC4- and IFI16-driven neuronal pyroptosis in SAH by modulating TLR4/NF-κB signaling. Pharmacological targeting of GPR30 represents a promising therapeutic strategy to ameliorate SAH-associated brain injury.

## 1. Introduction

Subarachnoid hemorrhage (SAH) is a life-threatening cerebrovascular disorder, typically characterized by sudden-onset severe headache [1]. Epidemiological data indicate an annual incidence of approximately 9 cases per 100,000 individuals, with SAH-associated mortality reaching 35% and a majority of survivors experiencing long-term neurological disability [2]. Immediate brain injury occurs immediately following the initial hemorrhage and the pathophysiological cascade within the first 72 h post-SAH—termed early brain injury (EBI)—has been identified as a critical contributor to delayed cerebral ischemia (DCI) and poor long-term functional outcomes in SAH patients [3]. Neurons are the primary cells responsible for rapid signal transmission in the brain. Their survival is critical for maintaining the stability and integrity of brain function and neuronal loss directly contributes to neurological deficits [4]. Following SAH, toxic substances (e.g., hemoglobin and iron ions) released from lysed red blood cells and inflammatory mediators spread through the neurovascular unit, driving secondary brain injury [5]. Neurons are particularly vulnerable to damage and are continuously exposed to post-SAH inflammatory responses, oxidative stress, and excitatory amino acid toxicity [6, 7]. Aberrant neuronal death and neuroinflammation are recognized as key contributors to EBI [8]. As a form of inflammatory cell death, pyroptosis emerged as a critical pathophysiological mechanism in EBI and its inhibition has been shown to effectively mitigate excessive inflammation post-SAH [9]. Thus, therapeutic strategies targeting neuronal pyroptosis hold significant promise for alleviating brain injury and neurological dysfunction after SAH.

The inflammasome, a critical mediator of innate immunity, is defined as a multiprotein complex that assembles following the detection of pathogen-associated molecular patterns (PAMPs) or damage-associated molecular patterns (DAMPs) by pattern-recognition receptors (PRRs) [10]. Inflammasome formation triggers caspase-1 activation, which subsequently cleaves gasdermin D (GSDMD) to induce membrane pore formation and pyroptotic cell death [11]. This GSDMD-dependent pyroptosis further amplifies the release of pro-inflammatory cytokines, including interleukin (IL)-1β and IL-18 [11]. Toll-like receptor 4 (TLR4), a key member of the TLR family, serves as a membrane-bound PRR critical for recognizing diverse PAMPs and DAMPs. TLR4 is widely expressed in the central nervous system (CNS), and TLR4-mediated signaling pathways are strongly implicated in neuroinflammatory dysregulation and clinical outcomes following SAH [12]. Prior studies demonstrate elevated TLR4 levels post-SAH, with TLR4 suppression attenuating cerebral edema and neuronal apoptosis [13, 14]. During EBI, TLR4-dependent neuronal apoptosis predominantly involves the TLR4/myeloid differentiation primary response protein 88 (MyD88) pathway, which drives nuclear factor kappa-B (NF-*κ*B) activation and subsequent upregulation of inflammatory mediators [15]. Notably, TLR4 has been shown to initiate inflammasome assembly and pro-IL-1β transcription via NF-κB activation [16]. Consequently, targeting the TLR4/NF-κB axis to modulate inflammasome activity and pyroptotic pathways may represent a promising therapeutic strategy for mitigating EBI after SAH.

G protein-coupled receptor 30 (GPR30), also termed G protein-coupled estrogen receptor 1 (GPER1), is a membrane-associated estrogen receptor. Emerging studies highlight GPR30′s significant role in modulating lymphatic vascular development [17], colorectal cancer progression [18], and hippocampal-dependent cognitive functions [19]. Notably, GPR30 demonstrates neuronal colocalization, with its expression markedly upregulated in SAH rats during EBI [20]. Peng et al. [20] further demonstrated that pharmacological activation of GPR30 using the agonist G1 reduced post-SAH neuronal apoptosis, whereas the antagonist G15 exacerbated cellular damage. Nevertheless, the mechanistic relationship between GPR30 and inflammasome activation via TLR4/NF-κB signaling in EBI pathogenesis remains unexplored.

Therefore, our study systematically evaluated the therapeutic effects of G1 and G15 in both SAH-induced rodent models and hemin-stimulated neuronal cultures. We aimed to elucidate the regulatory interplay between GPR30 and the TLR4/NF-κB/inflammasome axis in SAH pathophysiology.

## 2. Methods

### 2.1. Animals and SAH Model

Eighty male Sprague Dawley (SD) rats (280–330 g, 6–8 weeks old) were obtained from Hunan SJA Laboratory Animal Co., Ltd. (Changsha, China). Ten rats were randomly assigned to the sham group, while the remaining 70 rats underwent SAH induction. Rats failing to survive SAH (30% mortality rate) were excluded, yielding 49 SAH survivors. These were randomized into six experimental groups (*n* = 8/group). Eight rats from the sham cohort (*n* = 10) were similarly selected for downstream analyses.

The SAH model was established via intravascular perforation [21, 22]. In brief, rats were anesthetized with intraperitoneal pentobarbital (40 mg/kg), followed by surgical exposure of the left carotid artery. A tapered 4–0 nylon suture (Jinhuan Medical, Shanghai, China) was advanced from the external carotid artery (ECA) into the internal carotid artery (ICA) until perforating the bifurcation of the anterior cerebral artery (ACA) and middle cerebral artery (MCA). Immediate suture withdrawal permitted ICA reperfusion to induce SAH. Sham-operated rats underwent identical procedures excluding perforation. SAH severity was graded at 24 and 48 h postsurgery, followed by brain tissue collection.

To assess GPR30′s role, SAH rats received tail vein injections of the GPR30 agonist G1 (300 μg/kg; 881639-98-1, MCE, Monmouth Junction, NJ, US) or antagonist G15 (1 mg/kg; 1161002-05-6, MCE) dissolved in 10% dimethyl sulfoxide (DMSO), administered 1 h post-SAH [20]. Rats were euthanized via intraperitoneal pentobarbital overdose (200 mg/kg) and brain tissues were harvested for further analysis.

### 2.2. Short-Term Neurological Assessment

Neurological function was evaluated at 24 and 48 h post-SAH using the validated 18-point Garcia scoring system [23]. Scores were assigned based on six sensorimotor tasks, with higher values indicating superior neurological performance.

### 2.3. SAH Severity Grading

SAH severity was quantified at 24 and 48 h postinduction using a standardized scale [24]. The basal surface of the brain was divided into six regions, each graded 0–3 based on subarachnoid blood volume: 0: no hemorrhage, 1: minimal hemorrhage, 2: moderate arterial clot, and 3: severe clot obstructing all segmental arteries.

### 2.4. Cerebral Edema Measurement

Brain water content, a marker of cerebral edema, was determined via the wet–dry weight method [25]. Brains were rapidly extracted posteuthanasia (24/48 h post-SAH), bisected into hemispheres, and weighed to obtain wet weight (WW). Samples were dehydrated at 100°C for 24 h (oven Model 101-2EBS, Ever Bright Medical Treatment Instrument Co., Ltd., Beijing, China) and reweighed for dry weight (DW). Water content (%) was calculated as: [(WW − DW)/WW] × 100. Edema magnitude was defined as the difference in water content between left and right hemispheres.

### 2.5. SAH Cell Model

Rat brain neuronal cells (RAT-iCell-n005, iCell, Shanghai, China) were cultured in Dulbecco's modified eagle medium (DMEM) supplemented with 10% fetal bovine serum (FBS) and 1% penicillin–streptomycin. To establish the SAH model in vitro, cells were treated with hemin (H9039, Sigma–Aldrich, St. Louis, MO, USA) following established protocols [26]. Hemin was dissolved in sterile 0.1 M NaOH and further diluted in DMSO to prepare an 80 mM stock solution. The working solution was filtered through a 0.22 μm filter and applied to cells for 48 h.

For pharmacological interventions, neuronal cells were treated with G1 (0–100 μM) or G15 (0–10 μM) for 0–72 h to determine optimal dose- and time-dependent effects [27]. Additionally, TLR4 signaling was inhibited by cotreatment with 10 μg/mL LPS-RS (TLR4 antagonist; lipopolysaccharide from *Rhodobacter sphaeroides*, Invivogen, Carlsbad, CA, USA) [28].

### 2.6. Western Blot Analysis

Total protein was extracted using RIPA lysis buffer (AWB0136, Abiowell, Changsha, China). For membrane protein isolation, cells were homogenized in CER reagent with thorough vortexing. The lysate was centrifuged and the resultant supernatant was incubated with MER reagent. Membrane protein-enriched fractions were pelleted via ultracentrifugation, followed by resuspension in suspension buffer for downstream applications. Protein concentrations were quantified via a bicinchoninic acid (BCA) assay (AWB0104, Abiowell). Equal amounts of proteins were resolved on 10% SDS-PAGE and transferred to nitrocellulose (NC) membranes. Membranes were blocked with 5% nonfat milk in Tris-buffered saline containing 0.1% Tween-20 (TBST), followed by incubation with primary antibody (Table 1) at 4°C overnight. After three washes with TBST, membranes were incubated with horseradish peroxidase (HRP)-conjugated secondary antibodies for 90 min at room temperature. Protein bands were visualized using an enhanced chemiluminescence (ECL) substrate (AWB0005, Abiowell) and imaged on a ChemiScope6100 system (CLiNX, Shanghai, China). Band intensities were quantified using ImageJ software (NIH, Bethesda, MD, USA).

### 2.7. Immunofluorescence (IF) Staining

Neuronal cells were fixed with 4% paraformaldehyde, permeabilized with Triton X-100, and blocked with 5% BSA. Cells were incubated overnight at 4°C with the following primary antibodies: caspase-1 (1:50, 22915-1-AP, Proteintech), NLRC4 (1:50, ab201792, Abcam), IFI16 (1:50, ab169788, Abcam), and GSDMD (1:50, ab209845, Abcam). After washing, cells were stained with goat anti-rabbit IgG (H + L; 1:200, SA00013, Proteintech). Nuclei were counterstained with 4′, 6-diamidino-2-phenylindole (DAPI). Fluorescence images were captured using a BA410T microscope (Motic, China) at 100x and 400x magnifications. The percentage of positively stained cells was quantified using Image-Pro Plus software (Media Cybernetics, Rockville, MD, USA).

### 2.8. Cell Viability Assay (Cell Counting Kit-8 [CCK-8])

Cell viability was assessed using a CCK-8 (CK04, Dojindo, Kumamoto, Japan). Neuronal cells were seeded into 96-well plates (5 × 10^3^ cells/well). After treatment, 10 μL of CCK-8 reagent was added to each well, followed by incubation at 37°C with 5% CO_2_ for 4 h. Absorbance at 450 nm was measured using a microplate reader (MB-530, HEALES, Shenzhen, China).

### 2.9. Flow Cytometry

Pyroptosis was quantified using a FAM-YVAD-FMK/PI dual-staining assay. Staining solutions were prepared in the dark according to the number of experimental samples. Each sample was resuspended in 300 μL of washing buffer containing 2 μL of FAM-YVAD-FMK. After 1 h of incubation at room temperature in the dark, cells were centrifuged and washed twice. Propidium iodide (PI) working solution was added, followed by incubation at 37°C. Pyroptotic cells (FAM-YVAD-FMK+/PI+) were quantified using flow cytometer (A00-1-1102, Beckman, Miami, FL, USA) and FlowJo software (TreeStar, Ashland, OR, USA).

### 2.10. Transmission Electron Microscopy (TEM)

Hippocampal tissue samples were sequentially fixed in 2.5% glutaraldehyde and 1% osmium tetroxide. Dehydration was performed using a graded acetone series, followed by infiltration with incremental ratios of dehydration agent to Epon-812 epoxy resin. Samples were embedded in pure Epon-812 resin and polymerized. Ultrathin sections (60–90 nm thickness) were cut using an ultramicrotome (Leica, Germany) and mounted on copper grids. Sections were stained sequentially with uranyl acetate and lead citrate and examined under a transmission electron microscope (JEM1400, JEOL, Tokyo, Japan).

Neuronal specimens were fixed in 2.5% glutaraldehyde and 1% osmium tetroxide, followed by extensive washing with phosphate-buffered saline (PBS). Samples were dehydrated through a graded ethanol series, treated with a 1:1 (*v*/*v*) ethanol/isoamyl acetate mixture and incubated in pure isoamyl acetate for 1 h. After critical-point drying, specimens were sputter-coated with gold–palladium and imaged using a scanning electron microscope.

### 2.11. Statistical Analysis

Data are presented as mean ± standard deviation (SD). Normality and homogeneity of variance were verified using the Kolmogorov–Smirnov test and exploratory descriptive statistics test. For comparisons between two groups, unpaired two-tailed Student's *t*-tests were performed. One-way analysis of variance (ANOVA) with Tukey's post hoc test was applied for multigroup comparisons. All analyses were conducted using GraphPad Prism 9.0 software (GraphPad Software, San Diego, CA, USA). A *p*-value <0.05 was considered statistically significant.

## 3. Results

### 3.1. GPR30 Alleviates Neurological Impairment After SAH

**Figure 1:**
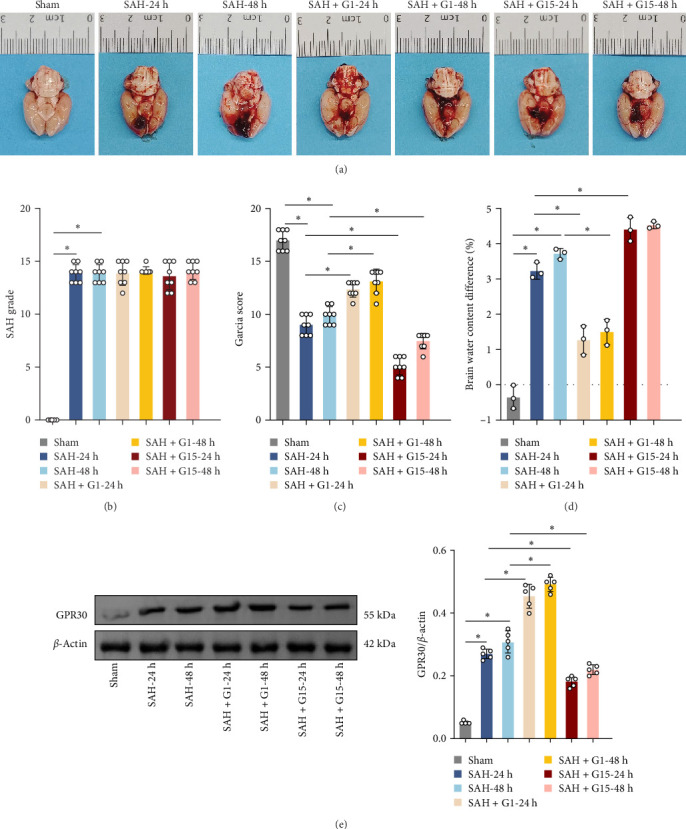
GPR30 agonist attenuates EBI after SAH. (A) Representative images of cerebral hemorrhage in rat brains. (B) Quantification of SAH severity grades across experimental groups. (C) Neurological function assessed by Garcia scoring system. (D) Brain water content difference (%) reflecting cerebral edema. (E) Western blot analysis of GPR30 protein expression levels. *⁣*^*∗*^*p*  < 0.05.

To investigate the regulatory role of GPR30 in SAH pathogenesis, rats were administered the GPR30 agonist G1 or antagonist G15 1 h post-SAH induction. Representative images of brain tissues at 24 and 48 h post-SAH revealed substantial intracranial blood clot formation (Figure 1A). Quantitative SAH grading confirmed severe hemorrhage in the SAH group (*p*  < 0.001 vs. sham), with no significant differences in hemorrhage severity observed between SAH, SAH + G1, and SAH + G15 groups (Figure 1B). Neurological function, assessed via the 18-point Garcia scoring system, demonstrated marked deficits in SAH rats compared to sham controls (*p*  < 0.001; Figure 1C). G1 treatment significantly improved neurological scores relative to the SAH group (*p*  < 0.001), whereas G15 administration exacerbated functional impairment (*p*  < 0.001 vs. SAH). Cerebral edema, quantified by bilateral hemispheric water content asymmetry, was elevated in SAH rats compared to sham animals (*p*  < 0.001; Figure 1D). This asymmetry was reduced by G1 (*p*  < 0.001 vs. SAH), but amplified by G15 (*p*  < 0.001 vs. SAH). Western blot analysis revealed upregulated GPR30 expression in the SAH group (*p*  < 0.001; Figure 1E). G1 further enhanced GPR30 expression (*p*  < 0.001 vs. SAH), while G15 suppressed its expression (*p*  < 0.001 vs. SAH). Collectively, these data indicate that GPR30 activation mitigates EBI post-SAH, whereas GPR30 inhibition aggravates neurological deficits and cerebral edema.

### 3.2. GPR30 Regulates Neuronal Pyroptosis After SAH

**Figure 2:**
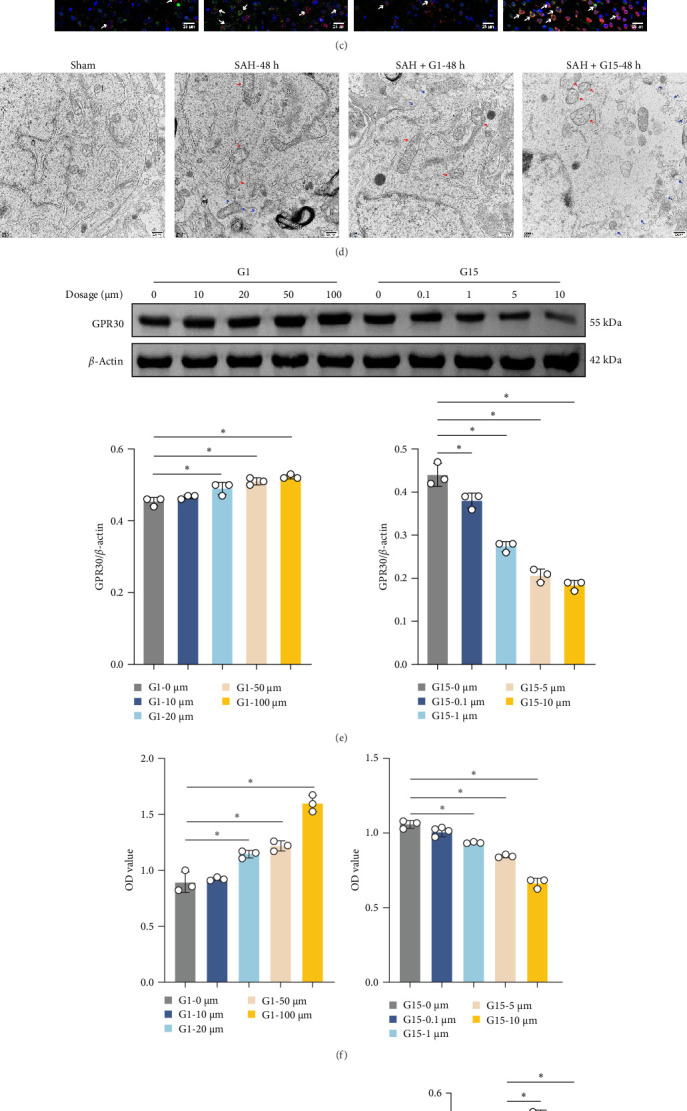
GPR30 regulates neuronal pyroptosis during SAH. (A) Protein expression levels of cleaved caspase-1, GSDMD-N, IL-1β, and IL-18 in brain tissues assessed by western blot. Immunofluorescence (IF) analysis of caspase-1+/NeuN+ (B) and GSDMD+/NeuN+ (C) colocalization in cortical brain tissue. White arrows denote cells exhibiting colocalization of the indicated markers. Scale bar = 50 μm (200x) or 25 μm (400x). (D) Transmission electron microscopy (TEM) images of pyoptotic neurons. Blue arrows indicate membrane rupture; red arrows denote swollen mitochondria with disrupted cristae. (E) Dose-dependent effects of G1 (0–100 μM) and G15 (0–10 μM) on GPR30 expression in neuronal cells using western blot. (F) Neuronal viability under G1 and G15 treatment measured by CCK-8 assay. (G) Quantitative analysis of GPR30 protein levels in neuronal cells. (H) Caspase-1-positive neurons quantified from IF images. Scale bar = 50 μm (up) and 25 μm (down). (I) Flow cytometric quantification of pyroptotic neurons. *⁣*^*∗*^*p*  < 0.05.

Pyroptosis, a caspase-1-dependent inflammatory cell death mechanism, contributes to secondary brain injury post-SAH [29]. Western blot revealed elevated protein levels of pyroptosis-associated markers, including cleaved caspase-1, GSDMD-N, IL-1β, and IL-18, in SAH rats compared to sham controls (Figure 2A, *p*  < 0.001). Pharmacological activation of GPR30 with G1 significantly suppressed these markers (*p*  < 0.001 vs. SAH), whereas GPR30 inhibition via G15 exacerbated their expression (*p*  < 0.001 vs. SAH). IF staining confirmed increased pyroptotic neuronal death in the cortical regions of SAH mice (Figure 2B,C). TEM revealed mitochondrial swelling and cell membrane rupture in the cortical regions (Figures 2D). Microglial overactivation is also considered a pivotal event in inflammation-mediated EBI [30]. We validated an increased proportion of microglial pyroptosis in cortical brain tissue post-SAH using IF staining (Supporting Information 1: Figure S1). These data highlight the importance of pyroptosis during SAH.

To further delineate GPR30′s role, in vitro experiments were conducted using neurons. Dose-response assays identified 100 μM G1 and 10 μM G15 as optimal concentrations for modulating GPR30 expression without cytotoxicity (Figure 2E,F; *p*  < 0.001). G1 induced a dose-dependent upregulation of GPR30 (*p*  < 0.001), while G15 suppressed its expression (Figure 2E, *p*  < 0.001). Neuronal viability, assessed via CCK-8 assay, increased with G1 treatment (*p*  < 0.001), but declined with G15 (Figure 2F; *p*  < 0.001). Time-course experiments demonstrated maximal GPR30 modulation at 48 h, with prolonged exposure (72 h) yielding no significant additional effects (Supporting Information 2: Figure S2A; *p*  < 0.001).

In a hemin-induced in vitro SAH model, GPR30 expression was upregulated in neurons (Figure 2G; *p*  < 0.001). G1 further amplified this response (*p*  < 0.001 vs. hemin), whereas G15 attenuated it (*p*  < 0.001). Hemin triggered caspase-1 activation, which was mitigated by G1 (*p*  < 0.001), but aggravated by G15 (Figure 2H; *p*  < 0.001). Flow cytometry confirmed that G1 reduced pyroptotic cell death in hemin-treated neurons (*p*  < 0.001), while G15 increased its incidence (Figure 2I; *p*  < 0.001). These findings demonstrate that GPR30 activation attenuates neuronal pyroptosis post-SAH, whereas its inhibition potentiates this inflammatory cell death pathway.

### 3.3. GPR30 Suppresses Inflammasome Activation After SAH

**Figure 3:**
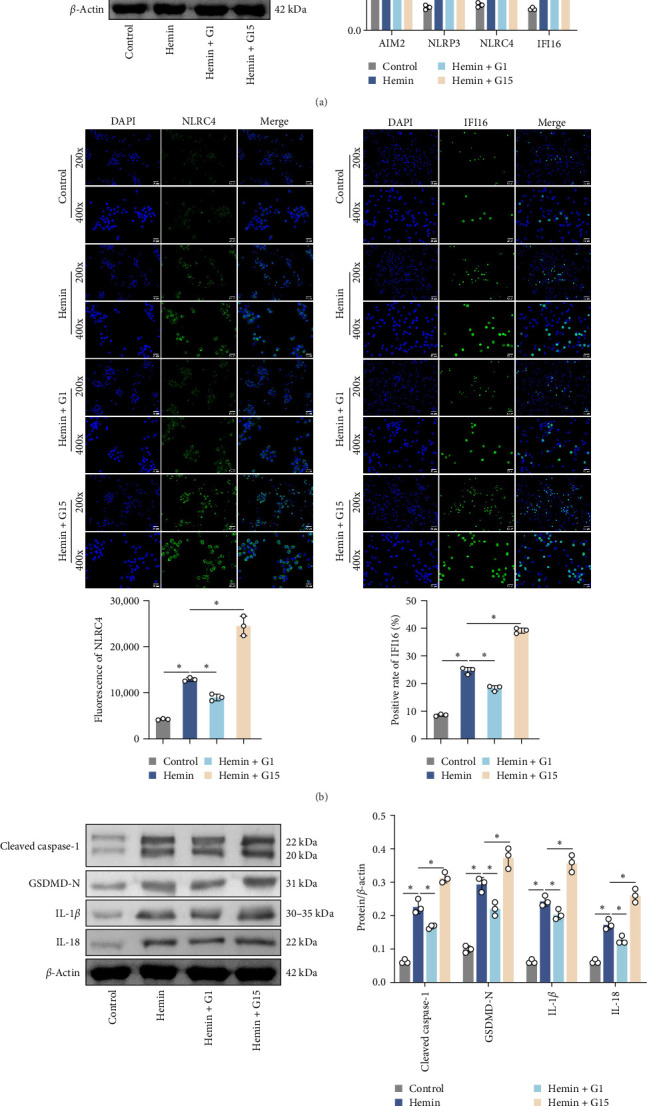
GPR30 modulates inflammasome activation after SAH. (A) Western blot analysis of inflammasome sensors AIM2, NLRP3, NLRC4, and IFI16. (B) IF staining of NLRC4 and IFI16 expression in neurons. Scale bar = 50 μm (up) and 25 μm (down). (C) Western blot analysis of cleaved caspase-1, GSDMD-N, IL-1β, and IL-18. *⁣*^*∗*^*p*  < 0.05.

Inflammasome assembly, a critical driver of pyroptotic signaling [31], was evaluated in SAH models by quantifying key inflammasome complexes (AIM2, NLRP3, NLRC4, and IFI16). Hemin-exposed cells exhibited marked upregulation of all four inflammasome components compared to controls (Figure 3A; *p*  < 0.001). Pharmacological inhibition of GPR30 with G15 amplified this response, significantly elevating AIM2, NLRP3, NLRC4, and IFI16 levels in hemin-treated cells (*p*  < 0.001). Conversely, GPR30 activation via G1 selectively reduced NLRC4 and IFI16 expression (*p*  < 0.001), with minimal impact on AIM2 and NLRP3. Given the pronounced modulation of NLRC4 and IFI16 by G1/G15, subsequent analyses focused on these two inflammasomes. IF staining corroborated that G15 intensified NLRC4 and IFI16 expressions in hemin-injured neurons, while G1 suppressed their expression (Figure 3B; *p*  < 0.001). Western blot further demonstrated that G1 attenuated hemin-induced cleavage of caspase-1 and GSDMD-N, as well as IL-1β/IL-18 maturation (*p*  < 0.001), whereas G15 exacerbated these pyroptosis-associated markers (Figure 3C; *p*  < 0.001). Collectively, these data indicate that GPR30 inhibits NLRC4- and IFI16-dependent inflammasome activation during SAH, while GPR30 blockade exacerbates this inflammatory cascade.

### 3.4. GPR30 Modulates TLR4/NF-κB Signaling in SAH Pathogenesis

**Figure 4:**
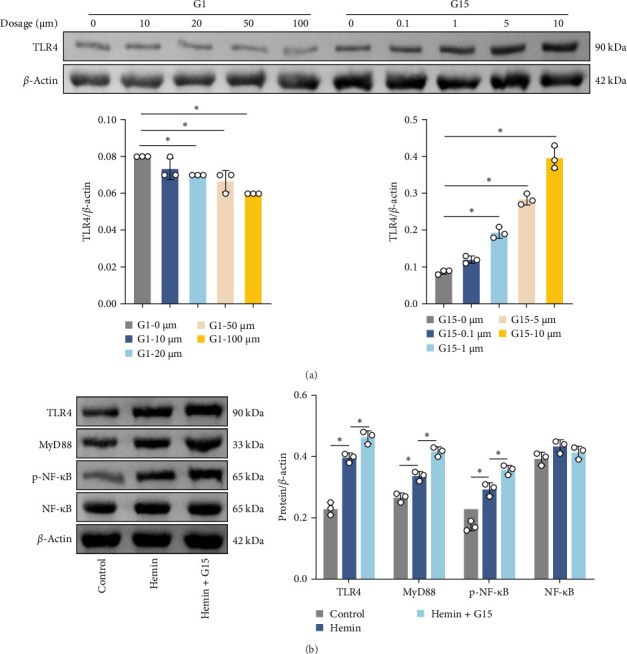
GPR30 suppression enhances TLR4/NF-κB signaling activation. (A) Dose-dependent effects of G1 (0–100 μM) and G15 (0–10 μM) on TLR4 expression in neuronal cells using western blot. (B) Western blot analysis of TLR4, MyD88, p-NF-κB, and NF-κB. *⁣*^*∗*^*p*  < 0.05.

To elucidate the regulatory role of GPR30 in TLR4/NF-κB signaling, dose–response studies were performed using the GPR30 agonist G1 and antagonist G15. Neuronal cells exposed to increasing concentrations of G1 exhibited progressive suppression of TLR4 expression relative to untreated controls (Figure 4A; *p*  < 0.001). Conversely, G15 administration induced a concentration-dependent upregulation of TLR4 in neuronal cells (*p*  < 0.001). G1 reduced TLR4 plasma membrane expression in neurons, whereas G15 upregulated TLR4 membrane localization (Supporting Information 2: Figure S2B; *p*  < 0.001). Given the robust TLR4 induction by G15, subsequent experiments utilized 10 μM G15 to evaluate downstream pathway components. Hemin exposure significantly elevated protein levels of TLR4, MyD88, and phosphorylated NF-κB (p-NF-κB) compared to controls (Figure 4B; *p*  < 0.001). GPR30 inhibition via G15 further amplified these effects, potentiating hemin-induced upregulation of TLR4, MyD88, and p-NF-κB (*p*  < 0.001). In contrast, total NF-κB protein abundance remained unchanged across experimental groups (*p*  > 0.05). These results demonstrate that GPR30 inactivation exacerbates TLR4/NF-κB pathway activation during SAH.

### 3.5. TLR4/NF-κB Signaling Drives Inflammasome Activation After SAH

**Figure 5:**
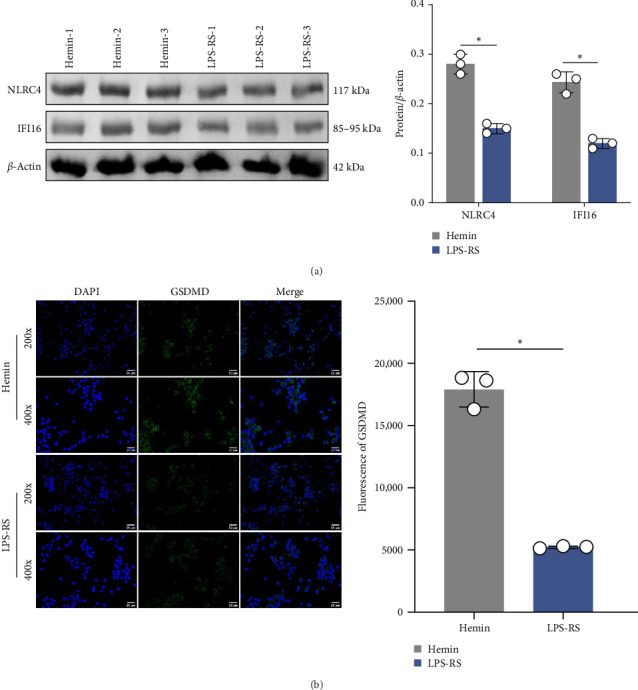
TLR4/NF-κB pathway inhibition suppresses IFI16 and NLRC4 inflammasome activity. (A) Western blot analysis of IFI16 and NLRC4. (B) IF analysis of GSDMD expression in neurons. Scale bar = 50 μm (up) and 25 μm (down). *⁣*^*∗*^*p*  < 0.05.

To investigate the mechanistic relationship between TLR4/NF-κB signaling and inflammasome assembly, SAH models were treated with the selective TLR4 antagonist LPS-RS. Pharmacological TLR4 inhibition via LPS-RS reduced IFI16 and NLRC4 protein relative to hemin-treated cells (Figure 5A; *p*  < 0.001). IF analysis demonstrated that LPS-RS suppressed hemin-induced GSDMD expression. These data collectively indicate that TLR4/NF-κB signaling is essential for IFI16 and NLRC4 inflammasome signaling.

### 3.6. GPR30 Governs TLR4/NF-κB-Dependent Inflammasome Activation

**Figure 6:**
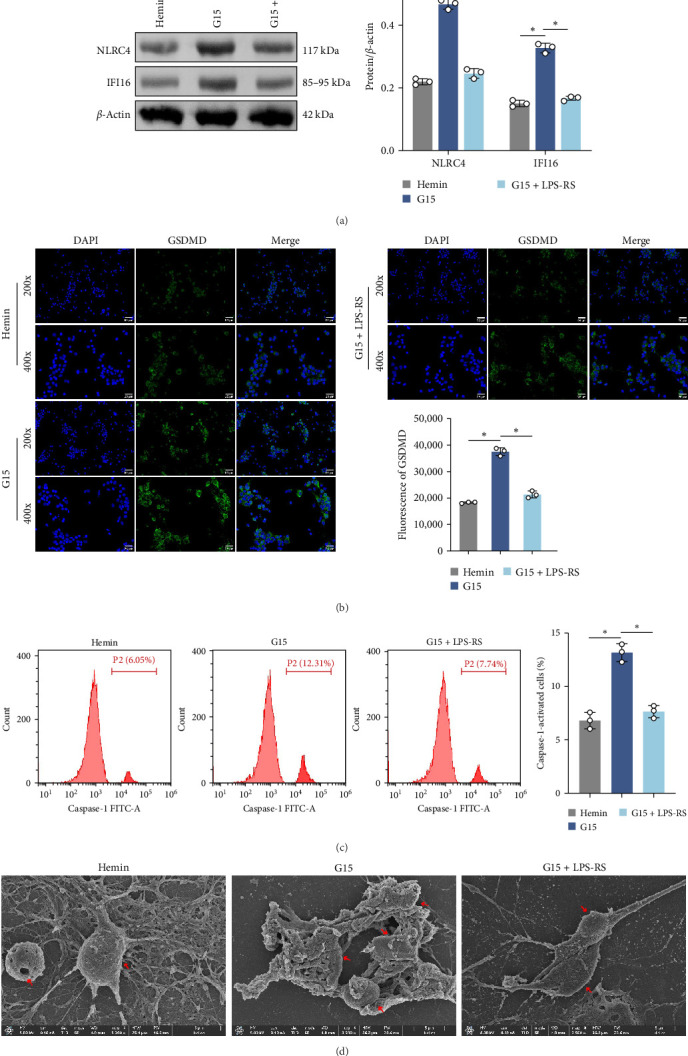
GPR30 silencing activates IFI16/NLRC4 inflammasomes through TLR4/NF-κB signaling. (A) Western blot analysis of IFI16 and NLRC4. (B) GSDMD expression visualized by IF staining. Scale bar = 50 μm (up) and 25 μm (down). (C) Pyroptosis rates quantified using flow cytometry. (D) TEM images of pyroptotic neurons. Red arrows indicate cytoplasmic vacuolization. *⁣*^*∗*^*p*  < 0.05.

To mechanistically interrogate whether GPR30 regulates IFI16/NLRC4 inflammasomes through TLR4/NF-κB signaling, SAH models were subjected to combined pharmacological modulation with G15 and LPS-RS. GPR30 inactivation via G15 significantly amplified hemin-induced IFI16 and NLRC4 protein accumulation (Figure 6A; *p*  < 0.001). Cotreatment with LPS-RS abolished this effect, reducing IFI16 and NLRC4 levels (*p*  < 0.001). IF analysis revealed that G15 enhanced GSDMD expression, this upregulation was attenuated by LPS-RS (Figure 6B; *p*  < 0.001). G15 and LPS-RS cotreatment group exhibited a reduction in pyroptotic cell death compared to G15 monotherapy (Figure 6C; *p*  < 0.001). Hemin induces neuronal cell swelling, membrane pore formation, and bubble-like pyroptotic bodies, with G15 further exacerbating cellular pyroptosis (Figure 6D). Collectively, these results establish that GPR30 silencing exacerbates SAH-associated IFI16 and NLRC4 inflammasome activation through TLR4/NF-κB signaling.

## 4. Discussion

The neuroprotective role of GPR30 has been extensively documented in prior studies [32–34]; however, its mechanistic involvement in EBI after SAH remains underexplored. Our data revealed a significant upregulation of GPR30 expression in SAH-induced rats. Pharmacological modulation further demonstrated that the GPR30 agonist G1 partially ameliorated neurological deficits and reduced cerebral edema/hemorrhage in SAH rats, whereas the antagonist G15 exacerbated brain injury and behavioral impairments. These findings collectively indicate that GPR30 overexpression confers neuroprotection against EBI post-SAH, while its inhibition aggravates pathological outcomes—a conclusion consistent with earlier reports [20].

Emerging evidence implicates GPR30 in suppressing TLR4/NF-κB signaling to mitigate neuroinflammation and ischemic injury [34]. Notably, ZEB1 has been shown to interact with GPR30, modulating inflammatory responses in ischemic stroke via TLR4/NF-κB pathway inhibition [35]. Our results align with these observations. G15 robustly increased TLR4 and MyD88 protein levels and NF-κB phosphorylation, confirming GPR30′s negative regulatory role in this pathway. The pathological significance of TLR4/NF-κB activation in SAH is well-supported. For instance, astragaloside IV attenuates post-SAH inflammation and vasospasm through TLR4/NF-κB suppression [36], while biglycan amplifies neuroinflammation by promoting microglial M1 polarization via TLR4/NF-κB activation [37]. Our work extends this paradigm by identifying GPR30 as a modulator of TLR4/NF-κB signaling in SAH pathology.

GPR30 may downregulate TLR4 in neurons through multiple mechanisms. First, GPR30 is predominantly localized to intracellular membranes (endoplasmic reticulum (ER) and Golgi apparatus) and the plasma membrane [38]. GPR30 could indirectly modulate TLR4 activity via G protein signaling crosstalk. Activation of GPR30 triggers Gαs-mediated adenylate cyclase stimulation, elevating cAMP/PKA signaling [39], which has been shown to inhibit TLR4/NF-κB activation [40]. Concurrently, GPR30 stimulates Gβγ subunit-mediated MAPK pathway activation [41]. Additionally, GPR30 has been reported to activate the PI3K/Akt pathway, mitigating blood–brain barrier dysfunction post-SAH [42]. Notably, both MAPK and PI3K/Akt pathways serve as secondary messengers modulating TLR4-driven inflammatory responses [43, 44]. Second, GPR30 may attenuate post-ischemic ER stress [45], thereby suppressing stress-induced TLR4 overexpression and associated neuroinflammation [46]. Finally, GPR30 might regulate TLR4 glycosylation, subcellular trafficking, and signaling via ER-Golgi-dependent mechanisms. TLR4 membrane localization requires N-glycosylation modifications processed in the ER and Golgi [47]. Prior studies implicate GPR30 in modulating glycosylation, maturation, and trafficking of membrane proteins [48].

The inflammasome system, particularly IFI16 and NLRC4, emerged as critical mediators in our study. IFI16, a cytosolic DNA sensor, orchestrates innate immune responses through inflammasome activation [49], with demonstrated roles in microglial-driven neuroinflammation [50]. Similarly, NLRC4 regulates bacterial defense mechanisms [51] and contributes to neuroinflammatory cascades in Alzheimer's models via IL-1β/Caspase-1/p-Tau axis activation [52]. Crucially, NLRC4 knockdown mitigates cerebral edema, neuronal death, and neurological impairment [53], mirroring our findings that G15 exacerbated IFI16/NLRC4 inflammasome accumulation in hemin-treated neurons, while G1 suppressed this response. Importantly, TLR4 inhibition abolished G15-driven IFI16/NLRC4 upregulation, establishing a mechanistic link between GPR30, TLR4/NF-κB signaling, and inflammasome regulation. This aligns with reports of GPR30-mediated NLRP3 inflammasome suppression in cerebral ischemia [54] and colitis models [55], though our study uniquely implicates IFI16/NLRC4 as key effectors in SAH.

Pyroptosis—an inflammasome-driven programmed cell death mechanism—was significantly modulated by GPR30 activity in our SAH model. G1 treatment reduced pyroptotic markers (cleaved caspase-1, GSDMD-N, IL-1β, and IL-18), whereas G15 amplified these effects. This aligns with established SAH pathophysiology, where inflammasome-driven pyroptosis exacerbates neuronal injury [56, 57]. Notably, TLR4 inhibitor (LPS-RS) reversed G15-induced pyroptosis and IFI16/NLRC4 activation, directly linking GPR30/TLR4 signaling to inflammasome-mediated cell death. Parallel mechanisms have been reported in Alzheimer's disease [58] and intestinal inflammation models [59] via TLR4/NF-κB/inflammasome inhibition. These findings underscore the broader relevance of TLR4/NF-κB/inflammasome pathway.

There are some limitations to the study. While pharmacological modulation (G15/G1) was employed to investigate GPR30′s role, the use of conditional GPR30 knockout mice would strengthen the mechanistic specificity of our findings. Genetic rescue experiments targeting TLR4/NF-κB or inflammasome components (IFI16/NLRC4) are warranted to confirm their functional necessity in GPR30-mediated neuroprotection post-SAH. The precise regulatory crosstalk between GPR30 and TLR4/NF-κB signaling during SAH merits in-depth exploration in future studies. While this study focused on neuronal pyroptosis, the broader role of GPR30 in neuronal and other neural cell death pathways post-SAH is essential.

In conclusion, our study delineates a novel GPR30-TLR4/NF-κB-inflammasome axis in SAH-induced EBI. Specifically, GPR30 downregulation exacerbates neuronal pyroptosis through IFI16/NLRC4 activation via TLR4/NF-κB signaling. These findings position GPR30 agonism as a promising therapeutic strategy to mitigate EBI following SAH.

## Figures and Tables

**Table 1:** Antibody information.

Antibody	Source	Dilution ratio	Manufacturer
GPR30	Rabbit	1:1000	ab260033, Abcam, Cambridge, UK
GSDMD-N	Rabbit	1:1000	ab215203, Abcam
Cleaved caspase-1	Rabbit	1:1000	4199, CST, Danvers, MA, USA
IL-1β	Rabbit	1:1000	16806-1-AP, Proteintech, Chicago, IL, USA
IL-18	Rabbit	1:5000	10663-1-AP, Proteintech
AIM2	Rabbit	1:5000	20590-1-AP, Proteintech
NLRP3	Rabbit	1:1000	19771-1-AP, Proteintech
NLRC4	Rabbit	1:1000	ab201792, Abcam
IFI16	Mouse	1:10,000	67790-1-Ig, Proteintech
TLR4	Rabbit	2 μg/mL	ab13867, Abcam
MyD88	Rabbit	1:5000	ab133739, Abcam
p-NF-κB	Rabbit	1:1000	ab76302, Abcam
NF-κB	Mouse	1:1000	66535-1-Ig, Proteintech
β-Actin	Mouse	1:5000	66009-1-Ig, Proteintech
ATP1A1	Rabbit	1:5000	14418-1-AP, Proteintech
HRP goat anti-mouse IgG	Mouse	1:5000	SA00001-1, Proteintech
HRP goat anti-rabbit IgG	Rabbit	1:6000	SA00001-2, Proteintech

## Data Availability

The datasets generated and/or analyzed during the current study are not publicly available, but are available from the corresponding author upon reasonable request.

## Supporting Information

Supporting Information 1 Figure S1: Immunofluorescence (IF) analysis of Caspase-1+/IBA1+ and GSDMD+/IBA1+ colocalization in cortical brain tissue. White arrows denote cells exhibiting colocalization of the indicated markers. Scale bar = 50 μm (200x) or 25 μm (400x).

Supporting Information 2 Figure S2: Effects of G1 and G15 on GPR30 and TLR4 expressions in neuronal cells. (A) Time–course optimization (0–72 h) of G1 (100 μM) and G15 (10 μM) treatment in neuronal cells. GPR30 expression was analyzed by western blot. (B) TLR4 expression in neuronal cells following 48-h treatment with G1 (100 μM) and G15 (10 μM), as assessed by western blot. *⁣*^*∗*^*p* < 0.05.
