# Supplementary figures and images for: G Protein-Coupled Receptor 30 Attenuates Neuronal Pyroptosis Induced by Subarachnoid Hemorrhage
Source: Mediators Inflamm. 2025 Jul 21;2025:3585885. doi: 10.1155/mi/3585885 (PMC12303633; doi:10.1155/mi/3585885)

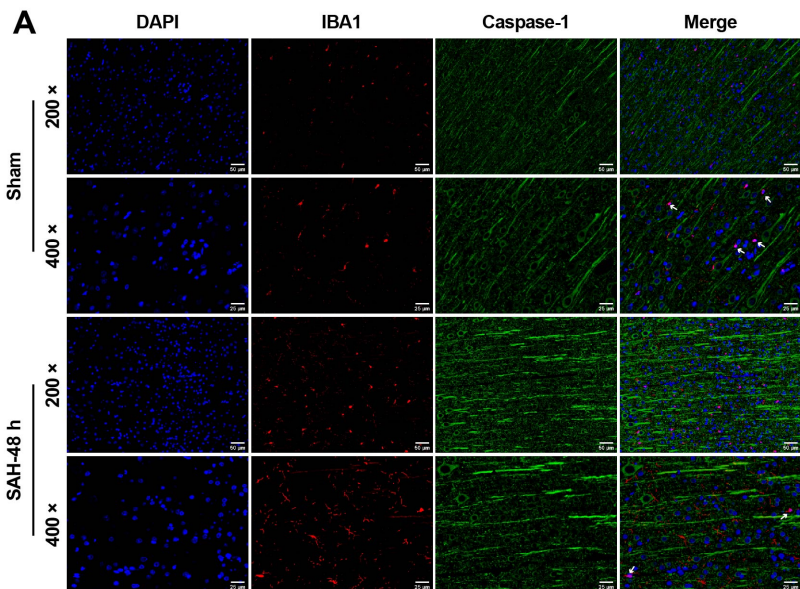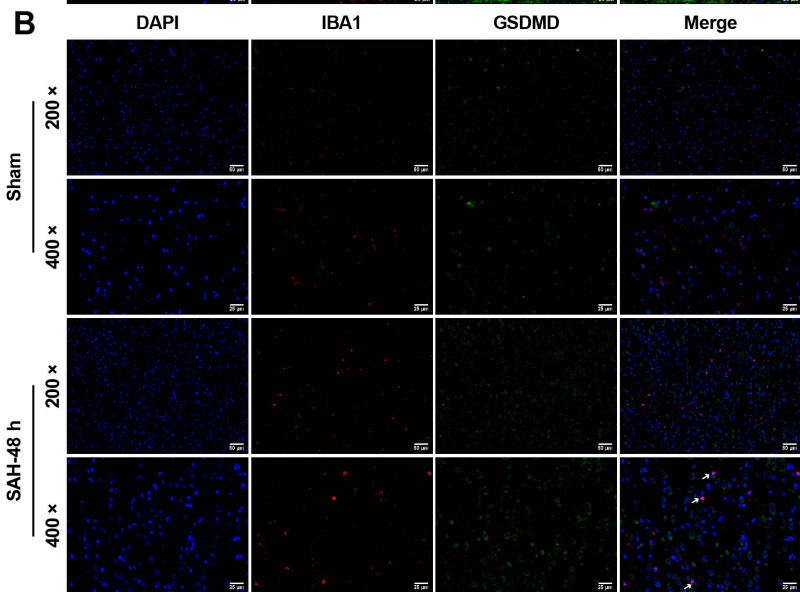

Supplement: Supporting Information 1 — Figure S1: Immunofluorescence (IF) analysis of Caspase-1+/IBA1+ and GSDMD+/IBA1+ colocalization in cortical brain tissue. White arrows denote cells exhibiting colocalization of the indicated markers. Scale bar = 50 μm (200x) or 25 μm (400x). [file 3585885.f1.pdf]

**A**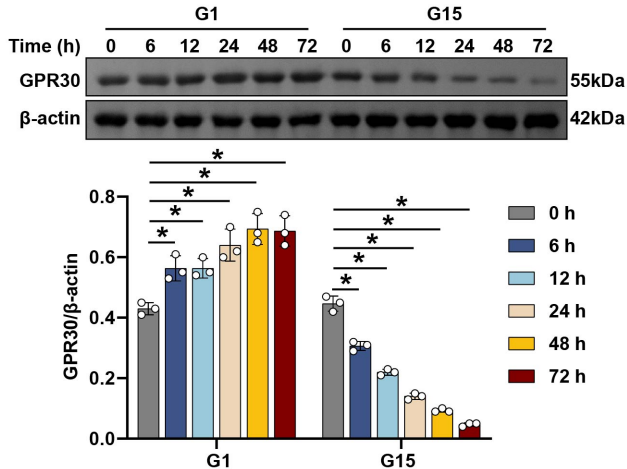**B**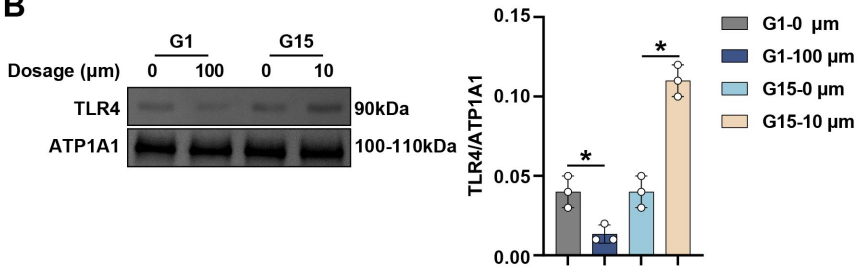

Supplement: Supporting Information 2 — Figure S2: Effects of G1 and G15 on GPR30 and TLR4 expressions in neuronal cells. (A) Time–course optimization (0–72 h) of G1 (100 μM) and G15 (10 μM) treatment in neuronal cells. GPR30 expression was analyzed by western blot. (B) TLR4 expression in neuronal cells following 48-h treatment with G1 (100 μM) and G15 (10 μM), as assessed by western blot. ⁣∗p < 0.05. [file 3585885.f2.pdf]
